# EEG Microstates Signatures of rTMS Response Over the lDLPFC: A Band-Specific Analysis

**DOI:** 10.1007/s10548-025-01146-7

**Published:** 2025-09-25

**Authors:** Marius A. Dragu, Gabriela Niculescu, Miralena I. Tomescu

**Affiliations:** 1 Department of Bioengineering and Biotechnology, National University of Science and Technology Politehnica Bucharest, Bucharest, Romania; 2 Department of Psychology and Educational Sciences, University Ştefan cel Mare of Suceava, Suceava, Romania; 3 Department of Research, Development and Innovation - CINETic, National University of Theatre and Film “I.L. Caragiale”, Bucharest, Romania; 4 Department of Psychology - Cognitive Sciences, University of Bucharest, Bucharest, Romania

**Keywords:** rTMS, iTBS, cTBS, EEG microstate, LDLPFC

## Abstract

Transcranial Magnetic Stimulation (TMS), particularly Theta Burst Stimulation (TBS), is a non-invasive, non-convulsive neuromodulation technique that induces clinically relevant network modulations with long-term effects. Two TBS protocols- continuous TBS (cTBS) and intermittent TBS (iTBS)- have been approved as effective therapeutic interventions for neuropsychiatric disorders, including mood disorders. With this aim, we examined EEG microstate temporal dynamics during resting-state recordings across three sessions of TMS. Twenty-four participants underwent cTBS, iTBS, and sham stimulation in a pseudo-randomized order, each separated by at least one week. Six distinct microstates (A–F), associated with activity in specific neural networks, were identified across six frequency bands (broadband, δ, θ, α, β, and γ). Our findings reveal frequency band-specific modulation of EEG microstates B, C, E, and F, previously reported as biomarkers in mood disorders. Notably, C microstates showed increased stability, whereas microstates E and F showed decreased dynamics up to fifty-five minutes after TBS. Most importantly, a negative association was observed for microstate E occurrence, between before stimulation (pre-cTBS) and three post-standing time points (post1-cTBS, post2-cTBS, and post3-cTBS), suggesting that baseline microstate E characteristics may be related to individual variability in cTBS treatment response. These results further support the potential of TBS to induce clinically relevant neuroplastic changes, establishing a strong foundation for the development of band-specific EEG microstate markers for assessing treatment response and personalized closed-loop TMS-EEG protocols.

**Supplementary Information:**

The online version contains supplementary material available at 10.1007/s10548-025-01146-7.

## Introduction

Transcranial Magnetic Stimulation is a non-invasive and non-convulsive Food and Drug Administration (FDA)-approved procedure for stimulating the human brain, which can induce clinically relevant network modulations with significant potential to produce long-term effects (Cole et al. 2022; Huang et al. 2007). Traditionally, according to the pulse frequency, the effects of TMS can inhibit brain activity (low-frequency TMS) and enhance it (high-frequency TMS), respectively (Bahadori et al. 2025; Bashir et al. 2022). Theta Burst Stimulation is an alternative type of repetitive Transcranial Magnetic Stimulation (rTMS) protocol developed to modulate cortical activity (Corp et al. 2020; Do et al. 2018; Hamada et al. 2013; Huang et al. 2007; Jannati et al. 2017; López-Alonso et al. 2014). Over time, three different TBS protocols have been employed: iTBS, cTBS, and consecutive bilateral cTBS/iTBS (Corp et al. 2020; Do et al. 2018; Giam et al. 2021; Hamada et al. 2013; Jannati et al. 2017; López-Alonso et al. 2014). More precisely, iTBS is delivered via three pulses (bursts), each at a typical frequency of 50 Hz, repeated at 200 ms intervals, equivalent to 5 Hz. In contrast, cTBS is applied continuously via a train of three pulses burst at 50 Hz (Chung et al. 2015). In general, it is believed that iTBS has an excitatory modulation effect on brain activity, while cTBS has an inhibitory effect; however, this contrast should be contextualized, as various studies highlight the intervariability in the behavioral and neurobiological TBS protocols (Corp et al. 2020; Do et al. 2018; Hamada et al. 2013; Jannati et al. 2017; López-Alonso et al. 2014). cTBS and iTBS may result in dissociable inhibitory versus excitatory effects due to interactions between the stimulation protocol, network configurations, the cytoarchitecture of brain regions, and disease pathophysiology, as supported by clinical data. Additionally, recent research indicates that the dissociable effects of TBS vary considerably depending on which prefrontal areas are stimulated, and the advantages of each patterned TBS procedure may vary depending on the clinical condition being treated (Kirkovski et al. 2023; Upton et al. 2023).

The FDA has approved several rTMS clinical applications developed for patients with various neuropsychiatric disorders (Davila-Pérez et al. 2018; Vucic et al. 2023). Prior investigations have demonstrated that individuals with neuropsychiatric disorders exhibit alterations in the prefrontal cortex, particularly in the left dorsolateral prefrontal cortex (lDLPFC). Modulation of DLPFC might have positive effects in neuropsychiatric disorders such as depression, schizophrenia, attention deficit hyperactivity disorder, mood and anxiety, post-traumatic stress disorder, to name a few (Li et al. 2022). DLPFC is a key node of the frontal cognitive circuit of attention (Bidet-Caulet et al. 2015; Vossel et al. 2014), decision-making (Rahnev et al. 2016), working-memory (Lin et al. 2022), and emotion regulation (Berboth and Morawetz 2021; Chen et al. 2023). Given its crucial role in cognitive functions and its proven effectiveness as a clinical target for major depressive disorder (Perera et al. 2016) by modulating plasticity (Chung et al. 2015), the DLPFC is one of the most frequently targeted brain regions in neuromodulation therapies (Kirkovski et al. 2023). Clinical trial protocols for depression have utilized iTBS on the left dorsolateral prefrontal cortex (lDLPFC) (Cole et al. 2022; Hemmings et al. 2024), characterized by hypofrontality (Spironelli et al. 2020) and reduced frontolimbic functional connectivity (Wackerhagen et al. 2020). At the same time, continuous cTBS on the right DLPFC (rDLPFC) has shown effectiveness in treating generalized anxiety disorder (Kirkovski et al. 2023). Notably, despite encouraging results in the clinical treatment of depression and comorbid disorders like anorexia nervosa and borderline personality disorder (Hemmings et al. 2024; Konstantinou et al. 2021), information on the neurocognitive mechanisms and individual variability in treatment response of lDLPFC TBS modulation is lacking. Electroencephalography (EEG) is an effective technique for assessing the time-dependent spatiotemporal dynamics of resting-state networks, given its millisecond temporal resolution. Microstate analysis is a spatiotemporal approach that describes the fast-changing activity-correlated networks across different neuro-cognitive and clinical mental states (Catrambone and Valenza 2023; Chivu et al. 2024; Deolindo et al. 2021; Hill et al. 2023; Metzger et al. 2024; Michel and Koenig 2018; Sikka et al. 2020; Tarailis et al. 2024; Tomescu et al. 2024a; Zanesco et al. 2021). EEG microstates (MSs) exhibit brief patterns of stable electrical activity, characterized by positive and negative voltages, that last tens to hundreds of milliseconds (60–120 ms) before shifting to another temporarily stable topography, which are highly reproducible within and across subjects (Michel and Koenig 2018). Between four (A-D) and seven (A-G), such MSs have been identified; their orientation is illustrated in the Fig. 3a (Custo et al. 2017; Michel and Koenig 2018; Tarailis et al. 2024).

Several studies indicate that EEG microstates are the electrical signatures of networks in the resting state (Michel and Koenig 2018). In summary, MSs A-B are linked to visual and auditory/language sensory cortices, as well as arousal states. MS B is related to visual processing, including tasks involving self-related processes, and autobiographical memory with implications extending beyond visual stimuli and interacting with other microstates (Antonova et al. 2022; Bréchet et al. 2019; Michel and Koenig 2018; Milz et al. 2016; Seitzman et al. 2017; Tarailis et al. 2024). MSs C, D, and E have been linked to core regions of top-down functional networks such as DMN, DAN, and SN (Michel and Koenig 2018). MS C is involved in processing personally significant information, MS D is intricately associated with higher-order cognitive functions like working memory and attention (Antonova et al. 2022; Bréchet et al. 2019; Faber et al. 2017; Tarailis et al. 2024) whereas MS E is associated with interoceptive and emotional processing, involvement in salience and emotional significance detection (Deolindo et al. 2021; Michel and Koenig 2018; Schiller et al. 2020; Tarailis et al. 2024). MS F contributes to personally significant information processing and theory of mind (Bréchet et al. 2020; Custo et al. 2017; Tarailis et al. 2024) and, finally, MS G is thought a biomarker of physical well-being in the light of its association with the somatosensory network (Custo et al. 2017; Tarailis et al. 2024). Although these associations were previously investigated, definitive one-to-one associations are still under debate, and future studies are needed to clarify the neurogenerators of microstates.

Most importantly, by investigating their temporal dynamics, EEG MSs demonstrated its utility as a reliable biomarker of clinical states and various treatment responses in a fast-growing literature (Chivu et al. 2024; Michel and Koenig 2018; Tarailis et al. 2024; Tomescu et al. 2024b).

Only a few studies have investigated the modulatory effects of rTMS on the temporal dynamics of EEG MSs. These studies have yielded significant findings, providing a comprehensive understanding of the potential of rTMS in normalizing neural activity by modulating B, C, and D microstates. For example, Qiu et al. demonstrated that continuous theta-burst stimulation, applied for 40 s over the left motor cortex of twenty-eight right-handed healthy subjects, revealed a significant decrease in MS B frequency of occurrence (Hz) (Qiu et al. 2022). Croce et al. investigated the induced changes of rTMS in 16 healthy subjects. They found that the mean duration of MS C significantly increased after applying rTMS stimulation only to one core region of the DAN, namely the intraparietal sulcus, compared to the pre-stimulation period (Croce et al. 2018). Several other targets of the DMN, including the right and left angular gyri and the right temporoparietal junction, were not modulated. In a clinical context, Gold et al. 2022 showed rTMS induced similar modulations of MS C in addition to the downregulation of MS D after a six-week TMS protocol on EEG microstate dynamics in 49 patients with treatment-resistant depression (Gold et al. 2022). The TMS protocol consisted of 10 Hz–5 Hz frequency stimulation on the left DLPFC, administered over 30 sessions within a 6-week period, followed by six additional sessions. In another study, rTMS to the left lateral parietal cortex in Alzheimer’s disease patients was associated with significant changes, including an increased average mean duration of MS C parameters and a higher frequency of transitions from MS D to MS C in Parkinson’s disease patients (Hanoglu et al. 2022). In line with these observations, Zhang et al. demonstrated that TBS administered over the cerebellum in stroke patients upregulated C, as opposed to downregulating the D microstate temporal parameters (Zhang et al. 2024). Patients in the iTBS group received iTBS therapy once a day, consisting of five 5-minute sessions, five times a week, over a period of four weeks. Following this treatment, the parameters for MS D in the iTBS–routine rehabilitation training group were lower. In contrast, MS C parameters were higher than those of patients receiving only routine rehabilitation treatment (Zhang et al. 2024). However, these studies investigated only broadband changes and did not examine induced changes in MS dynamics within specific spectral bands.

This study addresses a gap in the literature by investigating the effects of TBS on EEG microstate dynamics in healthy individuals, assessing its potential as a biomarker for treatment response and long-term effects. By analyzing spatiotemporal microstate changes before and after TBS, we aim to clarify the neural mechanisms underlying TBS-induced neuroplasticity. In particular, we examine inter-individual variability in treatment response, which is crucial for optimizing TBS protocols for neuropsychiatric disorders and advancing personalized, more effective treatment strategies.

## Methods

### Dataset Description

In this study, we processed and analysed an EEG dataset acquired and reported by Moffa et al. (Moffa et al. 2022). More details about the database can be found at https://plus.figshare.com/collections/Dataset_supporting_the_study_Neuromodulatory_effects_of_theta-burst_stimulation_of_the_prefrontal_cortex_using_TMS-EEG_/5910329 (Moffa et al. 2022). The study employed a single-blinded crossover design and three types of transcranial magnetic stimulation (iTBS, cTBS, and sham) using a MagPro X100 with a 65 mm diameter Cool-B65 figure-8 stimulation coil applied tangentially over the F3 electrode, targeting the lDLPFC. To reduce bone-conducted auditory input, electrode movement, and post-pulse artifacts, the coil was placed at a 5 mm distance from the head. The cTBS protocol consisted of 600 pulses delivered over 40 s, whereas the iTBS protocol employed two-second trains of TMS, administered every 10 s, for 192 s (600 pulses). The sham condition involved placing an inactive coil on the head.

In the sham condition, to enhance auditory masking and maintain blinding in the sham condition, a secondary TMS coil was positioned approximately 20 cm behind the participant, oriented away from them. This coil delivered auditory stimulation, with its output intensity increased by 20% to compensate for the greater distance from the ears. It simulated either an iTBS or cTBS protocol, assigned randomly to half of the subjects in each group. Participants also received white noise via in-ear headphones at a volume sufficient to mask the TMS click sound, or as loud as was comfortable. Earmuffs were placed over the headphones to reduce auditory perception of the pulses during TMS-EEG blocks.

Resting motor thresholds (RMT) were used to determine stimulation amplitude. In this way, RMT was individually determined by identifying the lowest stimulation intensity that produced at least 3 out of 6 motor-evoked potentials (MEP) with a minimum peak-to-peak amplitude of 50 µV in the contralateral right first dorsal interosseous muscle. MEPs were recorded via electromyography (EMG) using a 1401 laboratory interface and 1902 amplifier (Cambridge Electronic Design, Cambridge, UK), with data acquisition performed through the Signal V4 software (Cambridge Electronic Design, Cambridge, UK). RMT assessment was conducted during the initial visit and was not repeated before subsequent stimulation sessions. The average RMT across participants was 65.9% (SD = 6.7) of the maximum stimulator output. Finally, stimulation intensity was set at 120% of each participant’s RMT for single-pulse TMS and 75% of RMT for TBS.

To investigate the potential of TBS as a therapeutic neuromodulation intervention for psychiatric and neurological diseases, high-density EEG data were collected before and after TBS. The dataset thus comprised 24 healthy participants, including 13 men and 11 women. All participants were right-handed, as determined by the Edinburgh Handedness Inventory. Individuals were excluded if they met any of the following criteria: a history of psychiatric or neurological conditions (including seizures or stroke), recent head trauma, current use of medications that could influence cognitive function, substance or alcohol abuse within the past three months, smoking, current pregnancy, or any contraindications for EEG procedure.

Participants had no previous psychiatric or neurological history. All participants provided written informed consent, and the experimental procedures complied with the institutional requirements of the University of New South Wales Human Research Ethics Committee. Table 1 shows the demographic statistics for the given group.

**Table 1:** Demographic data for the analysed dataset

Subject	Entire group	Men	Women
n	24	13	11
Age (range)	18–65	19–65	18–33
Age ($\:mean\pm\:$SD)	25.20$\:\pm\:$9.93	28.23$\:\pm\:$12.42	21.63$\:\pm\:$4
Education (years) (range)	12–22	12–22	14–20
Education (years) ($\:mean\pm\:$SD)	15.95$\:\pm\:$2.34	16.04$\:\pm\:$2.36	15.61$\:\pm\:$1.53

**Fig. 1:**
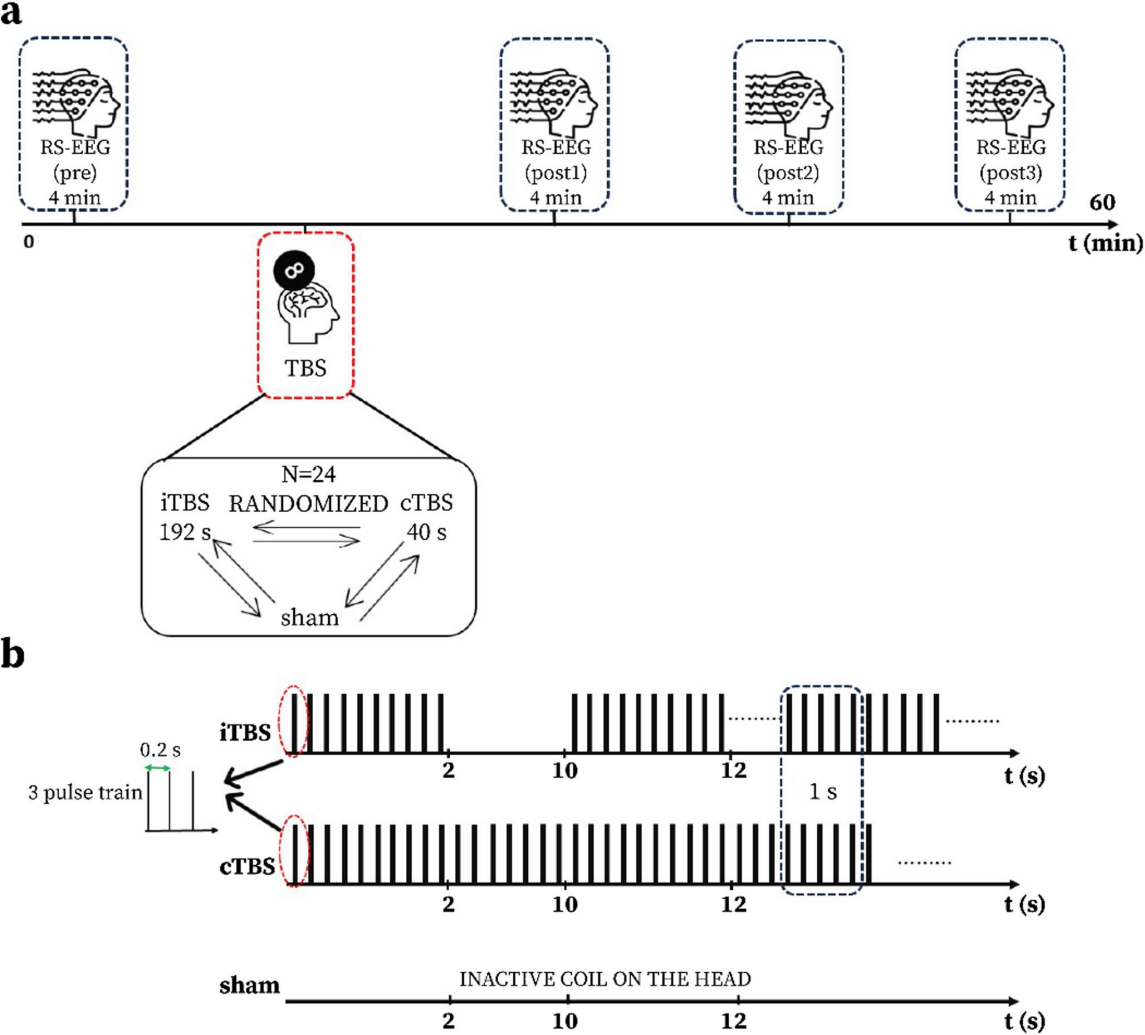
Pipeline resting-state acquisition and applied TBS protocols. (**a**) EEG recording pipeline. (**b**) TBS protocols: iTBS, cTBS, sham. iTBS is applied as a sequence of three pulses (= burst) at 50 Hz every 200 ms for 192 s. cTBS consists of applying continuous stimulation for 40 s. Sham involved placing an inactive coil on the head

The subjects participated in five different sessions in the lab, with at least one week between each session. Across the three sessions, the aforementioned three types of stimulation (cTBS, iTBS, sham) were applied, in a pseudo-randomized order (Fig. 1). Additionally, participants completed one session of iTBS and one session of cTBS. For the present analyses, only the first three sessions were considered.

Before (pre) and after (post) TBS, eyes open resting-state EEG signals (RS-EEG) were acquired for 4 min, using a 2048 Hz sampling frequency (sampling time = 0.48 ms), on 67 channels (64 primary and three auxiliaries: Vertical Electrooculogram, Horizontal Electrooculogram and Electrocardiogram) by a Refa 2048 EEG system (TMSi, Oldenzaal, the Netherlands, https://www.tmsi.com/*).* The electrodes were placed in the extended 10–20 distribution. Moreover, before and after TBS, single-pulse TMS-EEG, acquired after pre, post1 and post2, was used to investigate the neuromodulatory effects on cortical activity induced by cTBS and iTBS. Therefore, according to the pipeline acquisition depicted in Fig. 1, RS-EEG blocks (duration: 4 min) were recorded for each stimulation session: one before stimulation (pre) and three post-standing time points (post1- ~25 min after stimulation, post2- ~40 min after stimulation, post3- ~55 min after stimulation). Most importantly, according to the authors, interventions using iTBS and cTBS were well tolerated, and the most frequent adverse effect was minor headaches (*n* = 2, during iTBS and *n* = 1 during cTBS) (Moffa et al. 2022).

### Data Processing

**Fig. 2:**
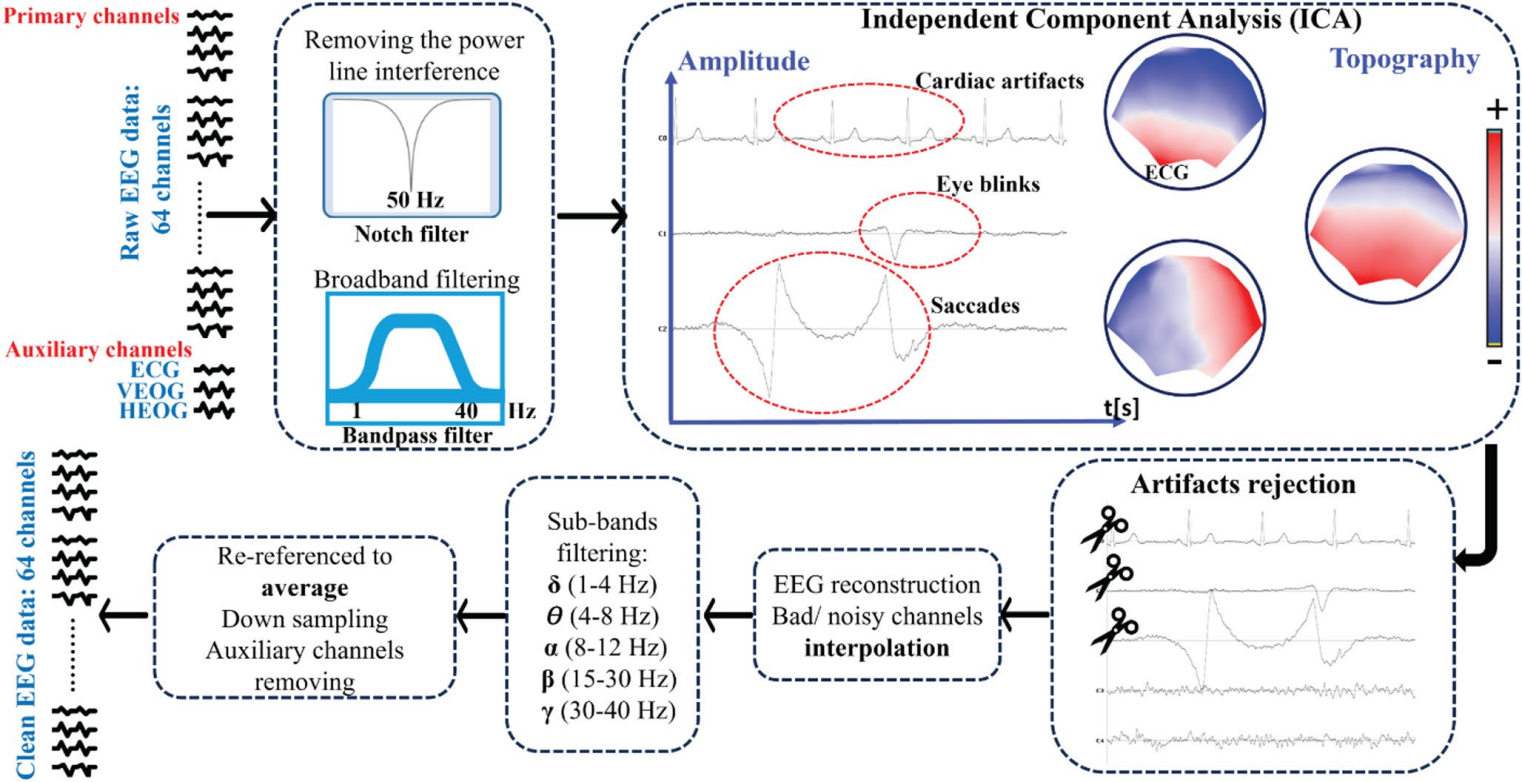
Flowchart of data processing: filtering (Using Notch and band-pass filters), computation of ICA, artifact rejection based on ICA, back-transform: brainwave reconstructions and interpolation of noisy channels, spectral band extraction (using a band-pass filter), and re-referencing to the average, downsampling, and removal of auxiliary channels

In the preprocessing stage **(**Fig. 2**)**, we filtered the raw data offline using a 50 Hz Notch filter to remove power line interference and a Butterworth band-pass filter between 1 and 40 Hz. Next, we applied the Infomax-based Independent Component Analysis (ICA) (Jung et al. 2000) Moreover, rejected eye blinks/saccades (eye movements) or cardiac artifacts (ECG) are identified based on visual inspection of the topography and amplitude of the components. Bad or noisy channels were subject to interpolation using a 3-D spherical spline (Perrin et al. 1989). Moreover, auxiliary channels were eliminated for each recording. Subsequently, we designed and applied four digital Butterworth bandpass filters to delimitate the EEG frequency bands [delta (δ, 1–4 Hz), theta (θ, 4–8 Hz), alpha (α, 8–12 Hz), beta (β, 15–30 Hz) and gamma (γ, 30–40 Hz)]. Finally, the initial database preprocessing was recomputed to the standard mean average and down-sampled to 128 Hz.

### Microstate Analysis

We employed microstate analysis to identify the most representative maps corresponding to the different microstates and to describe their temporal dynamics, mean duration, and occurrence. Microstates K-means clustering was applied to each frequency band (broadband, delta, theta, alpha, and beta) by participant and experimental condition (cTBS, iTBS, sham), resulting in the computation of the following methodology.

Firstly, we calculated the Global Field Power (GFP) (Lehmann and Skrandies 1980) defined for all electrodes as the standard deviation of the biopotentials of an average reference map (Mishra et al. 2020):

$$\:GFP\left({L}_{2}Norm\right)=\sqrt{\frac{\sum\:_{i=1}^{N}\left({V}_{i}\left(t\right)-\overline{V\left(t\right)}\right)}{N}},$$

where *V*_*i*_ is the voltage at channel *i* and $\:\overline{V}$ is the average voltage; N is the number of electrodes (specifically, *N* = 64).

The local maxima of *GFP (t)* show an optimal signal-to-noise ratio (Murray et al. 2008) in the EEG signals and represent ideal markers for determining quasi-stable topographic configurations. To find the most representative classes of stable topographies, the K-means algorithm was applied. Individual clustering level of EEG data is the first step in K-means clustering analysis. Therefore, the EEG signals were extracted at the corresponding time frames of GFP peaks. The GFP peaks were inputs for individual cluster-level analysis.

Next, to reduce computational time to obtain a representative set that captures sufficient variability in the data and forms a solid basis for the group-level clustering, we chose the same number of individual topographies across subjects—namely, seven. This choice was motivated by the maximum number of distinct microstate topographies (A–G) consistently reported in the literature (Custo et al. 2017; Tarailis et al. 2024). To automate the process and optimize runtime, based on the MNE Python library, we developed the code that provides the dominant topographies from each recording as a 7 (dominant topographies) x 64 (electrodes) array, corresponding to the centroids of the seven clusters with the highest Global Explained Variance (GEV) for each individual.

Then, to have a common reference for the back-fitting, the K-means algorithm was applied at the group level by clustering the 448 individual dominant topographies (from each frequency band). To establish the optimal number of clusters at the group level, we applied the criteria implemented in Cartool (an open-source academic software developed by Denis Brunet; cartoolcommunity.unige.ch), based on seven maximally independent criteria: Davies and Bouldin, Gamma, Silhouette, Dunn Robust, Point-Biserial, Krzanowski-Lai Index, and Cross-Validation (Brunet et al. 2011).

In the second part of the analysis, during the fitting process performed separately for each frequency band, the entire acquired and pre-processed EEG signal from subjects was used, excluding the marked artifact epochs. A temporal smoothing (window half-size 3 (24 ms), Besag factor of 10, and a rejection of small-time frames (when < 3, i.e., 24 ms) was applied. To determine the temporal parameters of microstates, every time point of the individual data was assigned to the microstate cluster with which it correlated the best (Brunet et al. 2011). A correlation coefficient threshold of 0.7 was applied to exclude short, transient noise frames in the EEG signal.

This fitting process enabled the description of topographical dynamics in terms of each subject’s duration and occurrence of each microstate. The duration (in ms) can be defined as the average continuous time that a given map is present, or the duration of the frames. The mean occurrence (in Hz) of a microstate is expressed as the rate at which a specific microstate was present, i.e., how many times per second that map was active. To gain a more fine-grained understanding, temporal dynamics analysis was carried out for each of the six frequency bands identified (broadband, δ, θ, α, β, and γ) (Férat et al. 2022).

The open-source academic software: Cartool (cartoolcommunity.unige.ch) and Python 3.9 were used for the EEG data processing and microstate analysis (Brunet et al. 2011; Gramfort et al. 2013).

### Statistical Analysis

We assessed data sets of microstate parameters, mean duration (ms), and occurrence (Hz), for normal distribution using the Shapiro-Wilk normality test and concluded that our data cannot be fitted to a Gaussian distribution. Subsequently, to investigate TMS-induced changes in the temporal dynamics of large-scale brain networks, we performed Wilcoxon signed-rank two-tailed tests and compared the results. We compared for each experimental condition (iTBS, cTBS, sham), the pre-TMS with each of the three consecutive post-TMS recordings, for each frequency band (broadband, δ, θ, α, β, and γ). We analyzed only within-condition time effects. To avoid potential confounds introduced by day-to-day variability, which could affect the reliability of direct comparisons, we did not perform between-condition comparisons, as each experimental condition took place on separate days.

We corrected for multiple comparisons using the false discovery rate (FDR) correction (Benjamini 1995). To quantify the size effect of the Wilcoxon signed-rank two-tailed tests, we performed the biserial rank coefficient (rb) (Kerby 2014). We evaluated the rb values according to McGrath and Meyer, where the interpretation of rb values is as follows: limited statistical (small) effect if rb < 0.10, medium if 0.10 ≤ rb ≤ 0.37, and evident (high) statistical effect for rb ≥ 0.37 (Fritz et al. 2012; McGrath and Meyer 2006).

Finally, we computed Spearman rank correlations between preTMS and change scores (postTMS-preTMS) microstate dynamics to assess inter-individual treatment response variability.

As correlation and regression are problematic for relating change scores to initial values due to mathematical coupling and regression to the mean, we tested preTMS–change correlations against the correct null hypothesis (i.e. the expected value of $\:{r}_{pre,post-pre}$) (Tu 2016; Tu and Gilthorpe 2007; Y.K. Tu et al. 2005).

## Results

### Topographycal

**Fig. 3:**
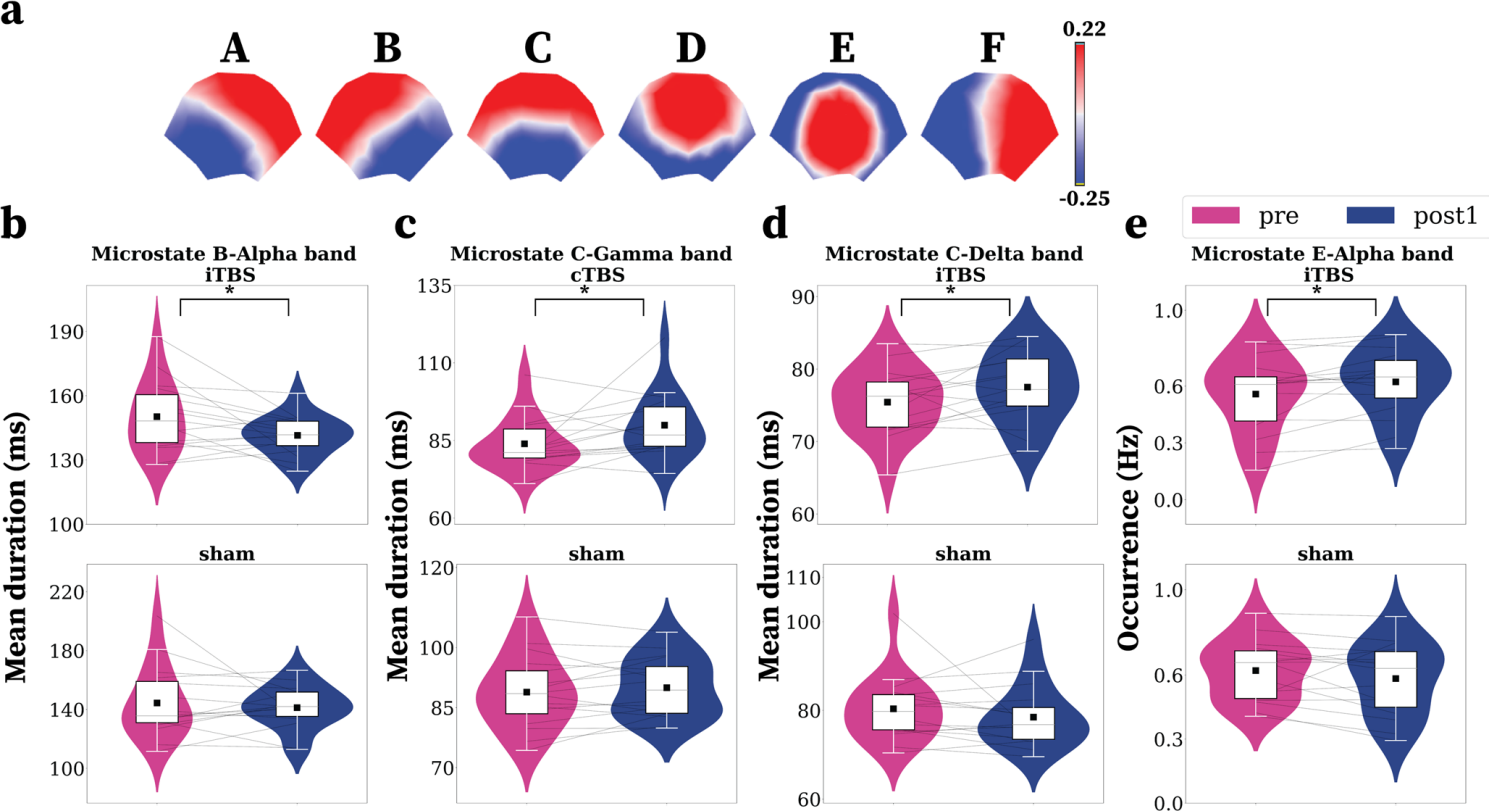
The microstate temporal dynamics (mean duration and occurrence rate) for short-lasting (10 min) effects: results for before and after the first recording. (**a**) Topographies of A to F MS (red: positive amplitudes, blue: negative amplitudes). MS A presents a left-right orientation, MS B exhibits a right-left orientation, MS C covers from frontal to occipital, MS D electric activity varies from centro-frontal to lateral and occipital and from centro-occipital to frontal in MS E, MS F with a left-lateralized maximum and MS G described by right-lateralized activity. (**b**) MS B (α band): Time effects results for mean duration (ms), iTBS, and sham. (**c**) MS C (γ band): Time effects results for mean duration (ms) for cTBS and sham. (**d**) MS C (δ band): Time effects results for mean duration (ms), iTBS, and sham. (**e**) MS E (α band): Time effects results for occurrence (Hz), iTBS, and sham. The grey lines, interconnecting the pre and post1 sessions, represent intra-individual data; the black squares represent mean values. In the boxplot (overlaid on the violin plot), the white square represents 50% of the data; the lower and upper boundaries correspond to the first (Q1) and third (Q3) quartiles, respectively, while the middle line represents the median value. The ends of the vertical white lines represent the values associated with Q1-1.5IQR and Q3-1.5IQR, respectively. The significant differences between inter-sessions are marked with FDR-corrected p-values: *p < 0.05

We found that six microstates best describe the group topographical variability, accounting for 84.85% of the explained variance (Fig. 3a), with high reproducibility and stability across individuals and frequency bands (Figure S1, Figure S2). The average spatial correlation between individual topographies for each frequency band and the group solution of six MSs ranges from 82% to 98%, with more than 80% exhibiting a spatial correlation higher than 0.92 **(Figure S2)**. Grand clustering for each frequency band reveals that, most often, we found six microstates as the optimum number of clusters (Figure S2a). The spatial correlation between the group clustering across all frequency bands and the separate clustering for each frequency band showed high values, ranging from 0.83 to 0.99, with a mean of 0.96 and a standard deviation of 0.04. **(Figure S2b)**.

To compare the temporal dynamics of time effects for each condition stimulation (iTBS, cTBS, sham), we ordered and labelled (A, B, C, D, E, F), according to the topographies reported in the literature (Custo et al. 2017; Michel and Koenig 2018; Tarailis et al. 2024).

### EEG Microstates Mean Duration (ms)

MS A, D, E, and F did not reveal significant time effects (pre vs. post1, pre vs. post2, pre vs. post3) in mean duration (ms) after the c/iTBS. However, we found short (25 min post1 iTBS and cTBS) and longer (55 min) cTBS effects across different MS B and MS C bands after TBS.

For **MS B**, in the ***α*** *band* we found a significant decrease (*p* = 0.039, r_b_=0.61, FDR corrected) of MS B mean duration during post1-iTBS (M ± SD: 141.5 ± 9.1) compared to pre-iTBS (150.2 ± 16.1) (Fig. 3b).

**MS C** showed a significantly higher mean duration (*p* = 0.039, r_b_=0.58, FDR corrected) in γ *band*, post1-cTBS (89.9 ± 10.4) compared to pre-cTBS (83.9 ± 8.4) and in ***δ*** *band* (*p* = 0.039, r_b_=0.61, FDR corrected), post1-iTBS (77.5 ± 4.8) than pre-iTBS (75.4 ± 4.6) (Fig. 3**c, d**). These effects lasted for approximately 25 min.

**Fig. 4:**
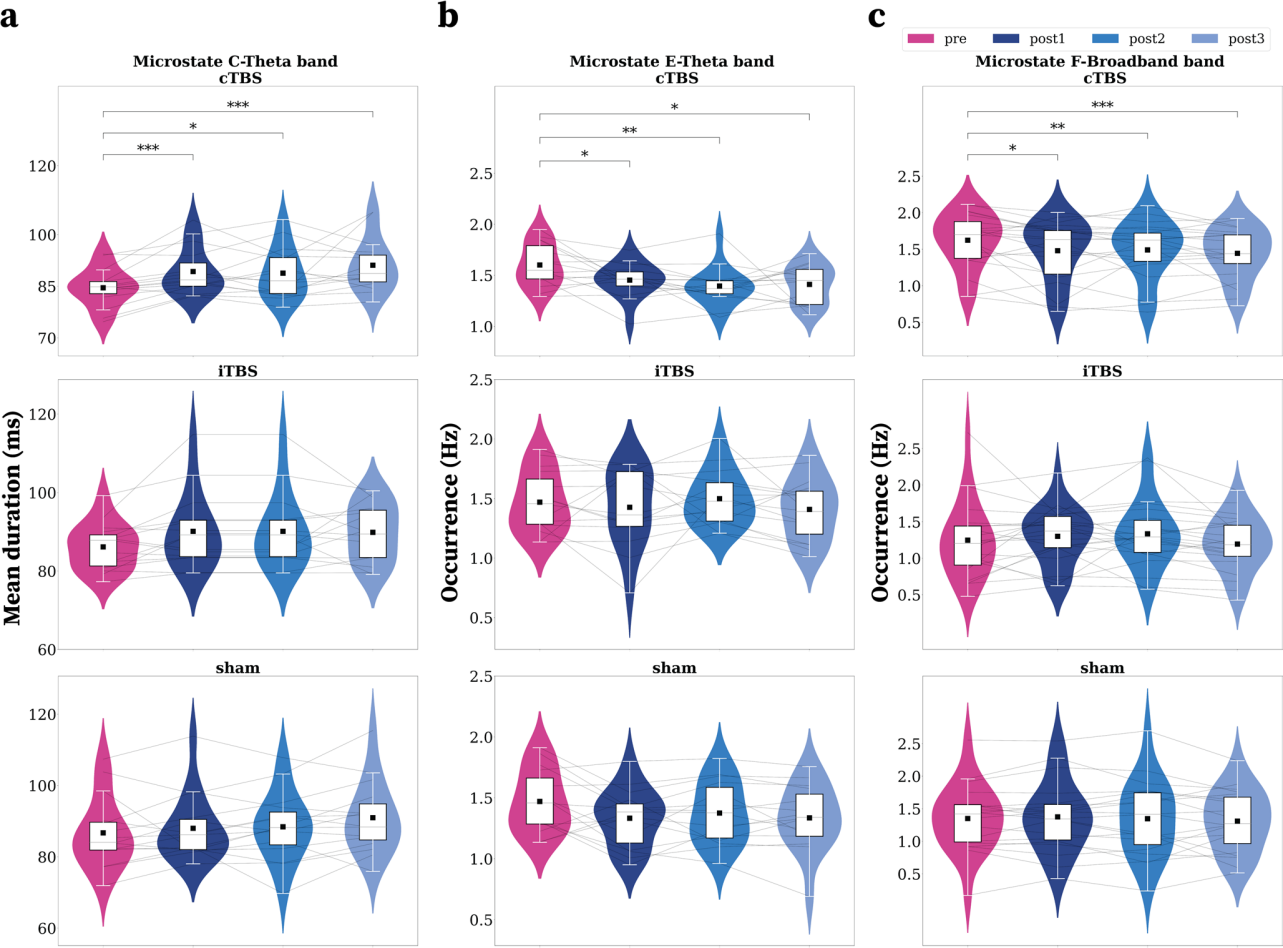
Time effects results (pre vs. post1, pre vs. post2, pre vs. post3) for microstates mean duration (ms) and occurrence (Hz). (**a**) Microstate temporal dynamics: MS C, results for the mean duration (ms) before and after TBS. (**b**) Microstate temporal dynamics: results for Occurrence (MS E, Theta Band). (**c**) Microstate temporal dynamics: results for Occurrence (MS F, Broadband). The grey lines, which interconnect multiple sessions, represent intra-individual data; the black squares represent the mean values. In the boxplot (overlaid on the violin plot), the white square represents 50% of the data; the lower and upper boundaries correspond to the first (Q1) and third (Q3) quartiles, respectively, and the middle line represents the median value. The ends of vertical white lines represent the value associated with Q1-1.5IQR and Q3-1.5IQR, respectively. The significant inter-session differences are marked with FDR corrected p-values *p < 0.05,**p < 0.01,***p < 0.001

Moreover, we found a significantly increased duration of **MS C** in the *θ band*, lasting 45 min until post3 after cTBS (Fig. 4a). There was a higher duration during post1 (*p* < 0.001, r_b_=0.88, FDR corrected), post2 (*p* = 0.036, r_b_=0.667, FDR corrected) and post3-cTBS (*p* < 0.001, r_b_=0.95, FDR corrected) compared to pre-cTBS (pre: 84.4 ± 5.4, post1: 88.7 ± 6.8, post2: 88.8 ± 7.4, post3: 91.1 ± 7.6). The mean duration results for all microstates (cTBS, iTBS, sham) are listed in **Supplementary Table ****S1**.

### EEG Microstates Occurrence (Hz)

MSs A, B, and D did not reveal significant changes in occurrence (Hz) after TBS. However, we found short-term modulations (25 min post1 iTBS and cTBS) for MS C and E, and long-term effects (55 min post-3 cTBS) across MS E and F following TBS.

**MS E**, in ***α*** band, occurs much more frequently in post1-iTBS than pre-iTBS (pre: 2.55 ± 0.19, post1: 0.6 ± 0.1, *p* = 0.022, rb = 0.67). These effects lasted for a short amount of time (10 min, Fig. 3e).

However, **MS E** in ***θ*** *band* exhibited a significantly reduced occurrence rate in post1-cTBS (*p* = 0.034, r_b_=0.61), post2 (*p* = 0.009, r_b_=0.83), and post3 (*p* = 0.014, r_b_=0.75) than pre-cTBS. The **MS E** cTBS effect lasted ~ 55 min (pre: 1.5 ± 0.2, post1: 1.3 ± 0.3, post2: 1.39 ± 0.19, post3: 1.4 ± 0.1) (Fig. 4b).

In addition, we found long-term effects ~ 55 min for **MS F** in *broadband* (Fig. 4c). There was a decreased occurrence in post1-cTBS (*p* = 0.028, r_b_=0.55, FDR corrected) post2-cTBS (*p* = 0.009, r_b_=0.67, FDR corrected) and post3 (*p* < 0.001, r_b_=0.81, FDR corrected) when compared to pre-cTBS. Indeed, after cTBS, MS F occurrence increased post1-cTBS, post2-cTBS and post3-cTBS than before cTBS (pre: 1.6 ± 0.3, post1: 1.4 ± 0.4, post2: 1.4 ± 0.3, post3: 1.4 ± 0.3). The occurrence rate results for all MSs (cTBS, iTBS, sham) are listed in **Supplementary Table S2**.

### Correlation Analysis

To assess individual variability in the cTBS response, we investigated whether the degree of cTBS modulation is associated with pre-microstate activity Thus, we computed the correlation between the post-pre MSs dynamics and the pre-MS activity considering the dynamics of **MS C** (mean duration, *θ band*), **MS E** (occurrence, *θ band*), and **MS F** (occurrence, *broadband*), for which the effects lasted for approximately 55 min. In addition, to rule out the possible impacts of mathematical coupling and regression to the mean, we computed the adjusted p-value, taking into consideration the correct null hypothesis (i.e., the expected values of $\:{r}_{pre,post-pre}$).

**Fig. 5:**
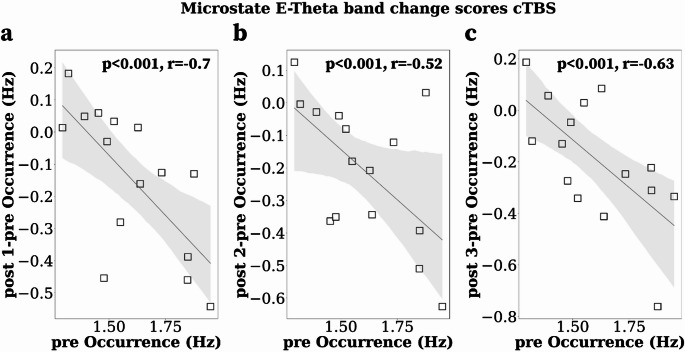
Spearman rank correlations between pre-cTBS MS E occurrence and change scores: post-pre cTBS MS E in the theta Band. (**a**) Correlation between pre and post1-pre occurrence. (**b**) Correlation between pre and post2-pre occurrence. (**c**) Correlation between pre and post3-pre occurrence

cTBS change scores for **MS E** in the *θ band* were significantly negatively correlated with baseline **MS E** pre-cTBS microstate dynamics (post1-pre, pre): *p* < 0.001, r_pre, post1-pre_=−0.7, r_pre, post1_ =0.25; (post2-pre, pre): *p* < 0.001, r_pre, post2-pre_ =−0.52, r_pre, post2_ =0.38; (post3-pre, pre): *p* < 0.001, r_pre, post3-pre_ =−0.63, r_pre, post3_ =0.34 (Fig. 5). The statistically corrected p-values suggest that pre-**MS E** correlates with the cTBS post-pre-**MS E**; this relationship cannot be explained by mathematical coupling or regression to the mean effects. The higher the baseline pre-cTBS **MS E** occurrence, the more significant the decrease in **MS E** during post1, post2, and post3 after cTBS.

Moreover, the correlation between baseline **MS E** pre-sham and post-pre-sham **MS E** occurrences were not significant (post1-pre, pre): *p* = 0.39, r_pre, post1-pre_=−0.25; (post2-pre, pre): *p* = 0.36, r_pre, post2-pre_=−0.26; (post3-pre, pre): *p* = 0.39, r_pre, post3-pre_=−0.21. For C and F MSs, we did not find significant correlations. Finally, the correlations between the C-E, C-F, and E-F cTBS change scores did not show significant associations.

## Discussion

This study investigated the TBS modulation on the temporal dynamics of band-specific EEG microstates in a within-subject crossover design. We found frequency-specific iTBS and cTBS mean duration (ms) and occurrence (Hz) significant modulations of B, C, E, and F MSs dynamics compared to sham. We found short-term effects (25 min) after TBS on **MS B**, **MS C**, and **MS E**. However, lasting effects were discovered approximately 55 min after 40 s of cTBS, lasting until the third recording. The dynamics of **MS C** increased, whereas the dynamics of **MS E** and **MS F** decreased after cTBS compared to sham and iTBS (Fig. 4). Moreover, we found that the magnitude of the cTBS response was associated with the individual variability of the baseline **MS E** dynamics (Fig. 5).

We identified patterns specific to the aforementioned frequency bands’ activity that might be concealed when we considered broadband analysis. For example, we observed significant changes in MS C durations when investigating time effects in delta, theta, and gamma, but not in alpha, beta, and broadband activity. According to Ferrat et al., this behaviour is potentially a consequence of the mutual cancellation of narrowband signals, inducing non-significant effects in broadband temporal dynamics analysis (Férat et al. 2022). Additionally, future studies should investigate how these findings relate to symptoms in the target population (e.g., depression).

Taken together, these EEG microstate findings across specific frequency bands further support the idea that band-specific EEG microstates can capture both intra- and inter-individual variability. This makes them promising candidates for biomarkers of treatment response and potential indicators of personalized treatment efficacy in TBS interventions.

### iTBS Effects on MS B

Our results show iTBS lDPFC down-modulated the mean duration of alpha (α 8–12 Hz) **MS B** (Fig. 3). **MS B** is one of the canonical microstates in the literature. It has been previously shown to change following cTBS on motor cortical areas, in association with improved contralateral hand dexterity in healthy individuals (Qiu et al. 2022). Thus, we further validate their findings that TMS protocols can modulate **MS B**; however, this time with iTBS on lDLPFC with possible clinical implications for mood and schizophrenia disorder patients.

### cTBS Effects on MS C

We found significantly short and prolonged mean durations of **MS C** on delta, theta, and gamma bands, lasting approximately 25 (short) and 55 (prolonged) minutes respectively, after TBS on the lDLPFC (Figs. 3 and 4). Our results align with the existing literature, which indicates that MS C exhibits increased dynamics after TMS applications when applied across various cerebral targets, including the inferior parietal sulcus and pre-supplementary motor area, using different stimulation protocols that encompass both high- and low-frequency stimulations, in both clinical and control populations. However, previous reports have shown changes in the topography of the MS C after TMS, which limit the validity of these interpretations. For example, Croce et al. reported that the **MS C** mean duration significantly increased after applying low frequency rTMS (for 1 min at 1 Hz) stimulation on the intraparietal sulcus compared to pre-stimulation (Croce et al. 2018). However, at the same time, the C topography changed into a topography resembling the **MS F**, making it difficult to accept that C increased after rTMS, which points more towards the idea that MS F explained more variance and replaced C when limiting the number of microstates to only four topographies. In addition, Hanoglu et al. reported a prolonged mean duration of **MS C** this time after high frequency rTMS (for 20–30 min at 5 Hz) on the pre-supplementary motor area in Parkinson’s disease patients (Hanoglu et al. 2022). However, their **MS C** topography after rTMS resembles the **MS E**, limiting this interpretation. More studies are needed to validate the **MS C** modulations after such different rTMS protocols.

An alternative explanation could be that **MS C** might be modulated through different connected brain regions, as it has often been associated with the large-scale resting-state network of the default mode network. More specifically, **MS C** is described as having many neural sources in posterior brain regions (the posterior cingulate cortex and the praecuneus, the left angular gyrus), but it has also been associated with prefrontal, anterior cingulate, and insular cortices (Britz et al. 2010; Custo et al. 2017). Moreover, the downstream effects of stimulating cortical regions should be taken into consideration. As Croce et al. argued, stimulating regions involved in the DAN, such as the intraparietal sulcus (and the left DLPFC, in our study), may indirectly modulate DMN-associated MS C, thereby demonstrating a causal link between these two anti-correlated networks. Alternatively, the regions targeted by the TMS stimulations that modulated **MS C** were part of the DMN network. Indeed, there is evidence in the literature that DLPFC modulations might regulate different networks based on the precise target location. As shown by Rosen et al., depression treatment response seems to be more specific to DLPFC-DAN stimulation than DLPFC-DMN stimulation (Rosen et al. 2021). However, in our study, we did not observe a modulatory effect of the **MS D** as others have reported after long-term use (six weeks) of high-frequency rTMS (Gold et al. 2022), usually associated with the DAN network and often observed anticorrelated to MS C modulations (Tomescu et al. 2022; Tomescu, et al. 2024a; Tomescu, et al. 2024b). Traditionally, cTBS is considered to produce inhibitory modulations and decrease functional connectivity in resting-state networks (Kirkovski et al. 2023). Thus, the prolonged **MS C** duration observed here may be a consequence of the downstream effects of reducing the inhibitory control of the DLPFC, *possibly a core region of* the DAN network, on the DMN, leading to an increase in **MS C** duration.

### cTBS Effects on MS E

**MS E** is one of the newly studied microstates associated with the salience network; however, when studying only four microstates, this microstate can be found to overlap with **MS C**. Custo et al. showed that these two microstates have different neuronal generators, with **MS E** being associated with activation in the dorsal anterior cingulate cortex, superior frontal gyrus, bilateral middle prefrontal cortex, and insular cortices, essential hubs of the salience network related to the processing of interoceptive and emotional information (Custo et al. 2017; Tarailis et al. 2024). Here, **the MS C** duration is increasing while the **MS E** occurrence is significantly decreasing in the theta band after cTBS on lDPFC, possibly suggesting that the opposite patterns of cTBS modulation within the same frequency band between **MS C** and **MS E** are due to compensatory functional roles, further supporting their distinct neural generators and functional roles. Alternatively, this might be explained by a causal effect of splitting similar MSs. Given that this effect was only visible in the theta band, these compensatory functional roles might sustain a reconfiguration of network dynamics associated with improved symptoms in patients; however, future studies are needed to disentangle these antagonistic patterns and their association with spontaneous cognition and clinical symptoms.

Most importantly, we observed that baseline **MS E** occurrence is associated with the degree of cTBS-induced modulation, with more substantial effects evident in individuals with higher pre-cTBS **MS E** occurrence. This suggests that pre-cTBS **MS E** can be seen as a potential biomarker of individual variability in cTBS responsiveness, although further studies are needed to confirm its predictive implication in cTBS treatment response.

### cTBS Effects on MS F

**MS F** is one of the newly studied microstates. It was proposed that MS F represents apart of the anterior DMN, playing a role in the processing of personally significant information, mental simulations, and the theory of mind (Bréchet et al. 2019; Custo et al. 2017; Tarailis et al. 2024). However, **MS F** is generally understudied in the microstate literature. As we are the first ones to observe post-TMS **MS F** occurrence reduction in broadband, more studies investigating more than four microstates are needed to confirm these results.

### Clinical Implications

**MS B** has been linked to autobiographical memory retrieval, spatial attention, and sensory networks (Bréchet et al. 2019; Tarailis et al. 2024), and is consistently increased in unmedicated mood disorder patients, possibly reflecting a compensatory mechanism involving visually guided rumination that exacerbates mood symptoms rather than alleviating them (Chivu et al. 2024). Our results suggest that iTBS over the lDLPFC may interrupt this maladaptive cognitive loop by reducing MS B-related retrieval processes, a hypothesis requiring further clinical validation in mood disorder populations (Kirkovski et al. 2023). Additionally, given similar MS B alterations observed in schizophrenia, spastic diplegia, and high-risk individuals (Andreou et al. 2014; Gao et al. 2017; Kikuchi et al. 2007; Michel and Koenig 2018), iTBS may offer transdiagnostic benefits by enhancing connectivity within the frontoparietal DAN network involved in visual mental representation and reward system regulation (Bation et al. 2021; Poorganji et al. 2023).

Increased **MS C** is typically observed during relaxed, self-referential states like mind-wandering, while decreased MS C occurs during cognitively demanding tasks (Antonova et al. 2022; Bréchet et al. 2019; Cui et al. 2021; Jabès et al. 2021; Katayama et al. 2007; Kim et al. 2021; Tarailis et al. 2024; Zappasodi et al. 2019). Our findings suggest that cTBS-induced modulation of **MS C** may promote more spontaneous cognition and reduce mind discontinuity, potentially benefiting neuropsychiatric conditions with diminished **MS C**, including dementia, panic disorder, stroke, borderline personality disorder, and anorexia nervosa (Berchio et al. 2024; Deiber et al. 2024; Kikuchi et al. 2007; Zappasodi et al. 2017), where rTMS over the lDLPFC has also been linked to BMI modulation (Bahadori et al. 2025; McClelland et al. 2016).

A decrease in **MS E**, often linked to increased **MS C** during mind-wandering, contrasts with its elevation during tasks requiring sustained attention or following emotional stress (Tarailis et al. 2024; Zanesco et al. 2021), and may reflect reduced alertness and emotional regulation after cTBS to the lDLPFC, consistent with fMRI findings of decreased SN connectivity post-rTMS (Godfrey et al. 2022). Notably, baseline **MS E** negative correlated with cTBS effects, with stronger modulation in individuals showing higher pre-stimulation **MS E**, a pattern associated with affective instability and altered **MS E** dynamics in borderline personality disorder (Deiber et al. 2024; Konstantinou et al. 2021).

## Limitations and Conclusions

By examining narrow-band EEG microstate modulations after cTBS and iTBS modulations compared to sham in a cross-over design over 24 healthy participants, we have established frequency band-specific modulation of EEG microstates (B, C, E, and F) previously shown to be altered in mood, schizophrenia, anorexia, and borderline personality disorder, to name a few. Although the study used a within-subjects design, the small sample size should be considered a limitation. Moreover, we can add the unique application dose and the mixed protocol of a single pulse of event-related potential TMS and resting-state data collection, which may have contributed to the post-TBS effects. Moreover, an important limitation of the present study is that no pre-post TBS measurements and correlation analyses with cognitive or perceptual parameters were conducted. Future studies should incorporate such measures to provide a more comprehensive understanding of the relationship between microstates and the cognitive processes potentially modulated by TBS.

However, we further confirm the potential of TBS to induce clinically relevant neuroplastic changes, providing a solid basis for the development of band-specific EEG microstate markers of treatment response, symptom monitoring, and personalized closed-loop TMS-EEG protocols. Future studies may also investigate the effects of TBS on longer time scales in neuropsychiatric patients after multiple-day interventions to confirm and monitor the normalizing effects in these patients. This would further investigate the intra-individual variability of treatment effects and shed light on the neuro-cognitive effects through experience sampling or questionnaire measurements of TBS modulations on spontaneous cognition.

## Supplementary Information

Below is the link to the electronic supplementary material.

Supplementary Material 1

## Data Availability

In this study, we processed and analysed an EEG dataset acquired and reported by Moffa et al. (Moffa et al., 2022). More details about the database can be found at https://plus.figshare.com/collections/Dataset_supporting_the_study_Neuromodulatory_effects_of_theta-burst_stimulation_of_the_prefrontal_cortex_using_TMS-EEG_/5910329 (Moffa et al., 2022).
