# Supplementary material for: EEG Microstates Signatures of rTMS Response Over the lDLPFC: A Band-Specific Analysis
Source: Brain Topogr. 2025 Sep 25;38(6):69. doi: 10.1007/s10548-025-01146-7 (PMC12464126; doi:10.1007/s10548-025-01146-7)
Supplement: Supplementary file 1 — Supplementary Material 1 [file 10548_2025_1146_MOESM1_ESM.docx]

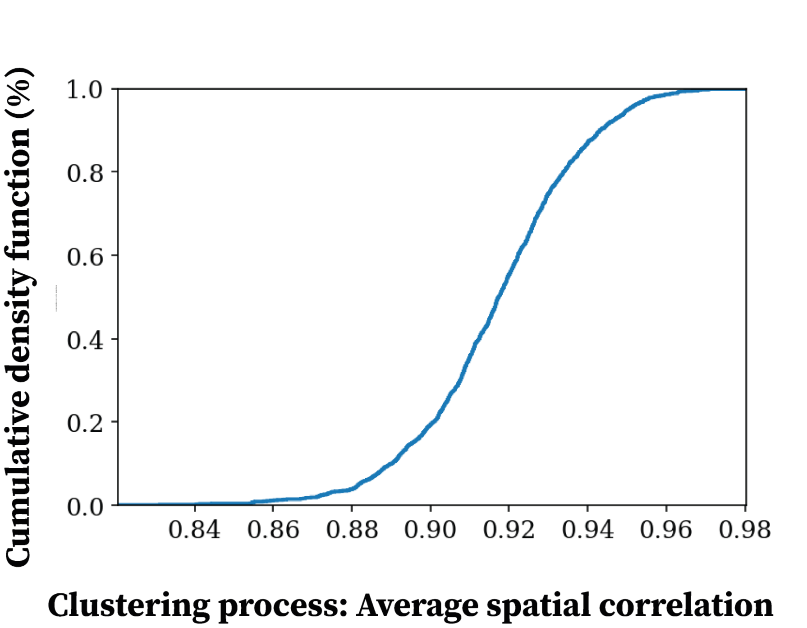


**Figure S1**: Cumulative density function of average spatial correlation between group-level clustering topographies and individual-level clustering topographies. The average spatial correlation was computed across the six group topographies and individual-level topographies for each frequency band (broadband, delta, theta, alpha, beta, gamma) and condition (cTBS, iTS, sham) (N = 6852 individual topographies).


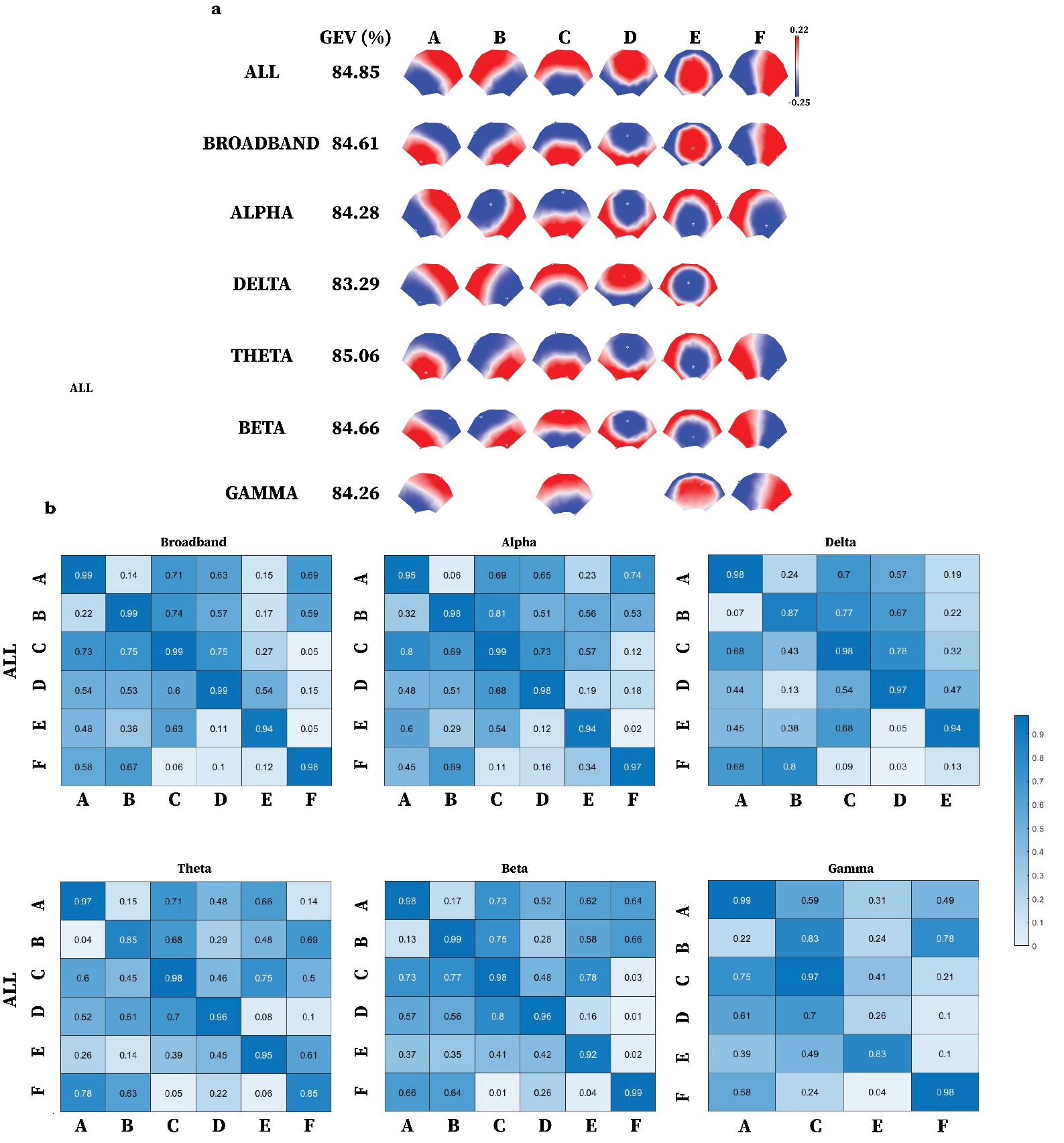


**Figure S2**: Clustering results across frequency bands. (a) Group-level clustering topographies: across all frequency bands (ALL) and separate for each frequency (broadband, alpha, delta, theta, beta, gamma) (b) The spatial correlation between ALL template topographies and template topographies for each frequency band.

**Table S1:** Mean-duration (ms) results (M ± SD) for each frequency band and time-point recording

| Broadband (1-40 Hz) cTBS | | | | | | |
| --- | --- | --- | --- | --- | --- | --- |
|  | MS A | MS B | MS C | MS D | MS E | MS F |
| pre | 89.03±10.65 | 77.41±5.42 | 78.01±4.05 | 76.39±3.88 | 71.69±3.67 | 72.96±4.85 |
| post1 | 88.74±1.23 | 77.99±7.48 | 77.56±6.63 | 75.72±3.96 | 72.77±4.89 | 72.01±4.98 |
| post2 | 90.49±13.72 | 77.56±6.36 | 77.46±5.29 | 76.77±5.61 | 72.67±4.34 | 71.85±3.91 |
| post3 | 93.38±14.37 | 76.62±6.73 | 78.64±6.44 | 76.65±5.41 | 72.45±5.15 | 70.69±4.77 |

| δ (1-4 Hz) cTBS | | | | | | |
| --- | --- | --- | --- | --- | --- | --- |
|  | MS A | MS B | MS C | MS D | MS E | MS F |
| pre | 66.32±2.32 | 67.16±3.45 | 78.24±5.46 | 67.29±3.99 | 67.73±2.46 | 62.06±4.18 |
| post1 | 68.31±3.14 | 66.54±3.67 | 79.31±6.41 | 69.31±2.96 | 67.37±4.85 | 63.31±3.03 |
| post2 | 67.53±2.54 | 67.31±3.42 | 79.14±6.68 | 68.15±3.47 | 68.76±2.57 | 62.96±3.39 |
| post3 | 67.29±3.03 | 67.33±3.23 | 81.61±9.48 | 68.61±3.59 | 67.44±3.41 | 62.37±3.22 |

| θ (4-8 Hz) cTBS | | | | | | |
| --- | --- | --- | --- | --- | --- | --- |
|  | MS A | MS B | MS C | MS D | MS E | MS F |
| pre | 74.41±4.18 | 74.49±4.28 | 84.44±5.45 | 74.84±8.01 | 63.37±4.12 | 66.28±4.21 |
| post1 | 76.25±4.58 | 76.38±4.43 | 88.78±6.86 | 77.69±11.48 | 63.22±6.33 | 67.63±4.85 |
| post2 | 77.27±3.18 | 76.72±5.08 | 88.81±7.43 | 78.06±10.25 | 64.87±4.31 | 67.51±4.08 |
| post3 | 77.64±3.79 | 77.63±4.79 | 91.11±7.69 | 79.25±9.85 | 63.81±4.66 | 67.41±4.91 |

| α (8-12 Hz) cTBS | | | | | | |
| --- | --- | --- | --- | --- | --- | --- |
|  | MS A | MS B | MS C | MS D | MS E | MS F |
| pre | 140.99±13.42 | 149.13±14.53 | 189.51±52.91 | 136.88±21.21 | 120.45±16.37 | 128.38±19.42 |
| post1 | 136.49±11.02 | 146.11±23.04 | 184.39±51.41 | 137.24±18.01 | 118.48±12.81 | 127.74±12.83 |
| post2 | 141.55±17.71 | 149.62±25.72 | 179.64±41.41 | 137.44±20.11 | 126.36±16.27 | 128.75±16.21 |
| post3 | 140.15±15.71 | 150.48±21.88 | 185.61±52.15 | 137.66±19.74 | 122.67±18.02 | 128.06±16.44 |

| β (15-30 Hz) cTBS | | | | | | |
| --- | --- | --- | --- | --- | --- | --- |
|  | MS A | MS B | MS C | MS D | MS E | MS F |
| pre | 80.48±3.77 | 79.77±3.51 | 87.07±6.95 | 75.86±3.77 | 71.11±6.91 | 74.93±4.61 |
| post1 | 80.98±2.62 | 80.12±4.70 | 86.76±5.67 | 75.73±2.93 | 69.31±4.71 | 74.48±3.34 |
| post2 | 80.95±4.03 | 79.67±3.83 | 85.25±4.92 | 74.48±3.51 | 68.39±4.06 | 74.51±3.38 |
| post3 | 81.08±4.34 | 80.20±3.52 | 87.22±8.95 | 74.30±3.89 | 67.66±5.41 | 74.72±4.52 |

| 𝛾 (30-40 Hz) cTBS | | | | | | |
| --- | --- | --- | --- | --- | --- | --- |
|  | MS A | MS B | MS C | MS D | MS E | MS F |
| pre | 78.62±5.22 | 78.53±5.53 | 83.91±8.43 | 71.41±4.47 | 67.85±8.81 | 74.56±5.76 |
| post1 | 80.95±4.92 | 79.81±6.78 | 89.92±10.42 | 71.05±4.19 | 69.05±5.13 | 75.18±5.29 |
| post2 | 81.39±6.97 | 81.01±8.91 | 87.83±10.71 | 69.27±4.07 | 68.24±6.13 | 76.15±6.11 |
| post3 | 81.23±6.96 | 79.16±6.77 | 88.91±11.38 | 71.52±5.55 | 68.79±4.75 | 74.95±7.27 |

| Broadband (1-40 Hz) iTBS | | | | | | |
| --- | --- | --- | --- | --- | --- | --- |
|  | MS A | MS B | MS C | MS D | MS E | MS F |
| pre | 85.24±12.27 | 71.44±5.28 | 73.54±5.37 | 72.58±5.49 | 67.42±4.64 | 65.55±3.79 |
| post1 | 84.01±9.32 | 73.31±6.89 | 73.79±4.09 | 72.74±3.71 | 68.44±3.64 | 65.93±4.21 |
| post2 | 87.73±14.42 | 73.66±7.06 | 74.31±5.31 | 73.99±5.48 | 68.68±4.55 | 67.82±5.33 |
| post3 | 89.95±12.72 | 72.89±6.49 | 75.58±5.52 | 73.81±4.43 | 68.55±3.91 | 66.21±3.68 |

*Cont.*

| δ (1-4 Hz) iTBS | | | | | | |
| --- | --- | --- | --- | --- | --- | --- |
|  | MS A | MS B | MS C | MS D | MS E | MS F |
| pre | 66.89±3.03 | 67.01±2.66 | 75.43±4.62 | 67.73±3.51 | 67.78±3.22 | 65.26±3.12 |
| post1 | 66.89±2.33 | 67.09±2.18 | 77.51±4.85 | 68.39±3.71 | 67.86±4.21 | 63.72±3.41 |
| post2 | 68.83±2.09 | 67.14±1.81 | 77.55±5.82 | 68.39±4.35 | 69.06±4.54 | 63.62±2.57 |
| post3 | 68.81±3.09 | 67.76±2.77 | 78.48±5.61 | 68.53±3.35 | 65.75±3.77 | 64.24±3.08 |

| θ (4-8 Hz) iTBS | | | | | | |
| --- | --- | --- | --- | --- | --- | --- |
|  | MS A | MS B | MS C | MS D | MS E | MS F |
| pre | 75.76-±4.82 | 77.24±4.62 | 86.02±5.85 | 75.78±7.57 | 63.41±5.15 | 68.36±5.02 |
| post1 | 76.97±4.13 | 77.09±4.37 | 87.23±5.96 | 78.16±12.19 | 63.81±5.81 | 69.41±4.53 |
| post2 | 76.37±5.17 | 76.84±5.84 | 90.13±9.57 | 77.71±9.19 | 65.31±8.35 | 69.59±4.71 |
| post3 | 77.23±3.72 | 77.13±4.58 | 89.88±7.44 | 79.13±8.87 | 65.44±5.12 | 69.51±4.63 |

| α (8-12 Hz) iTBS | | | | | | |
| --- | --- | --- | --- | --- | --- | --- |
|  | MS A | MS B | MS C | MS D | MS E | MS F |
| pre | 142.48±16.29 | 150.21±16.15 | 189.37±53.29 | 135.68±21.66 | 120.14±11.79 | 128.45±13.01 |
| post1 | 135.66±9.61 | 141.52±9.11 | 172.71±34.31 | 136.22±21.24 | 117.81±14.38 | 129.61±10.83 |
| post2 | 140.91±17.52 | 148.09±18.08 | 177.51±46.52 | 135.25±20.81 | 126.65±11.48 | 134.61±13.21 |
| post3 | 144.59±17.74 | 157.48±33.51 | 196.21±59.95 | 140.79±21.92 | 120.51±10.11 | 120.74±20.33 |

| β (15-30 Hz) iTBS | | | | | | |
| --- | --- | --- | --- | --- | --- | --- |
|  | MS A | MS B | MS C | MS D | MS E | MS F |
| pre | 80.64±4.04 | 79.18±4.74 | 84.51±6.01 | 74.91±3.63 | 68.01±5.82 | 74.44±3.62 |
| post1 | 79.81±2.42 | 79.11±3.57 | 83.97±3.33 | 74.99±3.81 | 68.33±5.79 | 74.12±3.11 |
| post2 | 80.22±3.66 | 79.61±4.62 | 85.91±6.11 | 74.15±3.48 | 68.40±5.10 | 74.80±4.61 |
| post3 | 81.61±4.13 | 80.82±4.56 | 86.46±7.83 | 75.44±3.67 | 67.30±4.52 | 75.44±4.12 |

| 𝛾 (30-40 Hz) iTBS | | | | | | |
| --- | --- | --- | --- | --- | --- | --- |
|  | MS A | MS B | MS C | MS D | MS E | MS F |
| pre | 77.68±5.51 | 75.93±5.35 | 80.11±9.66 | 68.47±3.88 | 62.94±4.88 | 73.34±7.38 |
| post1 | 77.46±5.78 | 76.75±5.73 | 81.86±7.66 | 68.72±5.21 | 64.86±5.55 | 73.08±4.74 |
| post2 | 76.89±6.03 | 75.71±5.57 | 82.84±7.83 | 67.95±4.81 | 65.35±4.01 | 73.46±6.19 |
| post3 | 78.53±7.16 | 77.09±5.42 | 83.68±9.68 | 68.61±3.94 | 63.77±4.53 | 73.86±6.19 |

| Broadband (1-40 Hz) sham | | | | | | |
| --- | --- | --- | --- | --- | --- | --- |
|  | MS A | MS B | MS C | MS D | MS E | MS F |
| pre | 73.81±4.44 | 73.16±6.26 | 89.25±15.03 | 71.09±10.21 | 67.61±4.98 | 66.61±4.33 |
| post1 | 75.77±5.26 | 73.68±6.51 | 92.71±22.02 | 72.65±6.08 | 68.21±4.04 | 67.72±4.03 |
| post2 | 73.76±4.91 | 72.27±6.68 | 91.06±18.32 | 72.41±7.91 | 66.41±4.5 | 67.92±3.87 |
| post3 | 73.79±4.31 | 74.98±5.42 | 94.94±22.95 | 73.00±8.28 | 67.24±6.11 | 67.67±3.05 |

| δ (1-4 Hz) sham | | | | | | |
| --- | --- | --- | --- | --- | --- | --- |
|  | MS A | MS B | MS C | MS D | MS E | MS F |
| pre | 69.12±2.12 | 66.94±4.83 | 80.36±7.53 | 67.84±8.46 | 67.28±3.94 | 63.06±3.68 |
| post1 | 70.41±4.51 | 64.91±4.57 | 78.47±6.97 | 67.08±8.16 | 67.31±3.45 | 64.47±3.84 |
| post2 | 70.22±3.45 | 65.58±4.41 | 79.41±6.81 | 67.96±7.95 | 66.82±4.84 | 64.71±4.13 |
| post3 | 68.78±2.72 | 67.11±4.32 | 80.12±6.34 | 68.63±5.46 | 68.91±3.59 | 63.39±3.21 |

*Cont.*

| θ (4-8 Hz) sham | | | | | | |
| --- | --- | --- | --- | --- | --- | --- |
|  | MS A | MS B | MS C | MS D | MS E | MS F |
| pre | 77.88±4.66 | 75.95±6.19 | 86.71±9.87 | 75.79±11.08 | 63.77±6.10 | 67.03±4.13 |
| post1 | 78.56±4.51 | 77.07±7.23 | 89.87±9.69 | 74.76±10.55 | 63.74±5.89 | 68.89±3.99 |
| post2 | 77.63±4.75 | 75.81±7.27 | 88.41±9.63 | 75.28±7.71 | 63.62±7.07 | 67.89±3.90 |
| post3 | 78.30±4.58 | 77.81±6.40 | 90.95±10.15 | 75.17±12.23 | 65.41±7.16 | 67.63±3.40 |

| α (8-12 Hz) sham | | | | | | |
| --- | --- | --- | --- | --- | --- | --- |
|  | MS A | MS B | MS C | MS D | MS E | MS F |
| pre | 142.94±16.47 | 144.42±24.20 | 185.10±38.95 | 137.77±27.40 | 122.81±18.46 | 127.82±17.50 |
| post1 | 146.75±13.98 | 141.22±14.56 | 182.81±34.81 | 137.21±24.74 | 116.71±0.27 | 129.78±12.83 |
| post2 | 145.24±17.38 | 142.29±12.56 | 187.41±51.09 | 128.11±25.38 | 120.48±20.47 | 131.62±16.06 |
| post3 | 143.37±15.05 | 155.18±21.12 | 192.94±62.36 | 135.36±24.46 | 118.97±16.73 | 132.22±16.37 |

| β (15-30 Hz) sham | | | | | | |
| --- | --- | --- | --- | --- | --- | --- |
|  | MS A | MS B | MS C | MS D | MS E | MS F |
| pre | 81.16±4.32 | 78.09±4.36 | 89.54±19.82 | 74.64±9.98 | 69.44±3.57 | 73.82±3.29 |
| post1 | 81.55±6.40 | 79.31±5.28 | 92.30±23.70 | 76.22±5.31 | 67.96±5.05 | 74.47±5.13 |
| post2 | 82.29±4.72 | 79.62±5.20 | 88.36±13.06 | 76.04±5.67 | 68.45±4.49 | 75.31±5.39 |
| post3 | 81.06±4.25 | 79.75±5.40 | 91.26±21.49 | 76.38±4.04 | 67.88±3.28 | 74.30±5.05 |

| 𝛾 (30-40 Hz) sham | | | | | | |
| --- | --- | --- | --- | --- | --- | --- |
|  | MS A | MS B | MS C | MS D | MS E | MS F |
| pre | 83.25±5.94 | 81.01±5.93 | 88.87±9.11 | 73.51±5.84 | 68.11±5.15 | 77.02±5.59 |
| post1 | 84.69±9.57 | 81.62±7.81 | 89.97±7.51 | 72.55±5.79 | 68.56±5.56 | 76.39±6.28 |
| post2 | 84.77±7.87 | 78.46±6.26 | 88.01±7.81 | 69.98±8.68 | 67.24±5.29 | 77.17±6.69 |
| post3 | 84.61±6.72 | 81.62±6.77 | 90.27±8.62 | 72.11±5.10 | 68.99±4.10 | 76.94±5.63 |

**Table S2:** Segment Density (Hz) results (M ± SD) for each frequency band and time-point recording

| Broadband(1-40 Hz) cTBS | | | | | | |
| --- | --- | --- | --- | --- | --- | --- |
|  | MS A | MS B | MS C | MS D | MS E | MS F |
| pre | 2.31±0.27 | 1.79±0.32 | 1.93±0.27 | 1.75±0.25 | 1.47±0.34 | 1.62±0.37 |
| post1 | 2.36±0.31 | 1.88±0.31 | 1.92±0.33 | 1.74±0.31 | 1.52±0.36 | 1.47±0.41 |
| post2 | 2.35±0.32 | 1.84±0.37 | 1.89±0.33 | 1.76±0.27 | 1.44±0.33 | 1.49±0.39 |
| post3 | 2.44±0.38 | 1.77±0.33 | 1.88±0.32 | 1.72±0.29 | 1.39±0.39 | 1.44±0.34 |

| δ (1-4 Hz) cTBS | | | | | | |
| --- | --- | --- | --- | --- | --- | --- |
|  | MS A | MS B | MS C | MS D | MS E | MS F |
| pre | 2.08±0.32 | 2.21±0.25 | 2.88±0.38 | 2.03±0.26 | 1.94±0.22 | 1.61±0.34 |
| post1 | 2.11±0.21 | 2.12±0.29 | 2.81±037 | 2.04±0.21 | 1.81±0.38 | 1.61±0.36 |
| post2 | 2.09±0.21 | 2.08±0.32 | 2.83±0.38 | 2.01±0.28 | 1.88±0.21 | 1.59±0.32 |
| post3 | 2.04±0.23 | 2.04±0.29 | 2.81±0.32 | 2.05±0.31 | 1.92±0.31 | 1.52±0.32 |

| θ (4-8 Hz) cTBS | | | | | | |
| --- | --- | --- | --- | --- | --- | --- |
|  | MS A | MS B | MS C | MS D | MS E | MS F |
| pre | 1.66±0.17 | 1.81±0.19 | 1.95±0.37 | 2.07±0.25 | 1.58±0.21 | 1.53±0.32 |
| post1 | 1.62±0.26 | 1.66±0.28 | 1.96±0.35 | 2.04±0.28 | 1.36±0.31 | 1.42±0.29 |
| post2 | 1.63±0.21 | 1.67±0.21 | 1.94±0.35 | 2.01±0.25 | 1.39±0.19 | 1.41±0.24 |
| post3 | 1.56±0.24 | 1.71±0.18 | 1.95±0.41 | 1.95±0.27 | 1.41±0.18 | 1.37±0.31 |

| α (8-12 Hz) cTBS | | | | | | |
| --- | --- | --- | --- | --- | --- | --- |
|  | MS A | MS B | MS C | MS D | MS E | MS F |
| pre | 0.92±0.11 | 1.02±0.21 | 1.22±0.19 | 0.75±0.21 | 0.62±0.17 | 0.74±0.21 |
| post1 | 0.92±0.19 | 1.07±0.19 | 1.22±0.17 | 0.79±0.19 | 0.61±0.18 | 0.79±0.25 |
| post2 | 0.95±0.19 | 1.06±0.17 | 1.21±0.21 | 0.79±0.18 | 0.63±0.15 | 0.79±0.19 |
| post3 | 0.93±0.18 | 1.04±0.19 | 1.22±0.22 | 0.76±0.17 | 0.59±0.18 | 0.75±0.23 |

| β (15-30 Hz) cTBS | | | | | | |
| --- | --- | --- | --- | --- | --- | --- |
|  | MS A | MS B | MS C | MS D | MS E | MS F |
| pre | 2.12±0.23 | 2.11±0.33 | 2.62±0.52 | 1.58±0.35 | 0.96±0.41 | 1.61±0.42 |
| post1 | 2.22±0.31 | 2.15±0.31 | 2.62±0.43 | 1.59±0.14 | 0.92±0.38 | 1.55±0.41 |
| post2 | 2.27±0.25 | 2.15±0.29 | 2.53±0.38 | 1.58±0.25 | 0.95±0.35 | 1.66±0.35 |
| post3 | 2.20±0.24 | 2.12±0.35 | 2.16±0.48 | 1.56±0.27 | 0.91±0.30 | 1.60±0.40 |

| 𝛾 (30-40 Hz) cTBS | | | | | | |
| --- | --- | --- | --- | --- | --- | --- |
|  | MS A | MS B | MS C | MS D | MS E | MS F |
| pre | 2.41±0.51 | 2.22±0.49 | 3.11±0.91 | 1.16±0.26 | 0.87±0.61 | 1.71±0.57 |
| post1 | 2.25±0.41 | 2.09±0.46 | 3.11±0.78 | 1.13±0.31 | 0.91±0.42 | 1.49±0.46 |
| post2 | 2.28±0.49 | 2.23±0.54 | 2.93±0.82 | 1.01±0.37 | 0.77±0.93 | 1.71±0.57 |
| post3 | 2.26±0.36 | 2.19±0.47 | 2.94±0.75 | 1.15±0.44 | 0.75±0.53 | 1.71±0.48 |

| Broadband (1-40 Hz) iTBS | | | | | | |
| --- | --- | --- | --- | --- | --- | --- |
|  | MS A | MS B | MS C | MS D | MS E | MS F |
| pre | 2.81±0.44 | 1.81±0.41 | 2.17±0.34 | 2.02±0.33 | 1.49±0.41 | 1.24±0.51 |
| post1 | 2.76±0.28 | 1.91±0.37 | 2.13±0.33 | 2.03±0.25 | 1.52±0.36 | 1.31±0.39 |
| post2 | 2.71±0.37 | 1.81±0.43 | 2.11±0.36 | 1.92±0.32 | 1.44±0.45 | 1.33±0.46 |
| post3 | 2.83±0.33 | 1.81±0.33 | 2.06±0.37 | 1.93±0.34 | 1.35±0.41 | 1.19±0.37 |

*Cont.*

| δ (1-4 Hz) iTBS | | | | | | |
| --- | --- | --- | --- | --- | --- | --- |
|  | MS A | MS B | MS C | MS D | MS E | MS F |
| pre | 2.14±0.26 | 2.16±0.23 | 2.68±0.31 | 2.06±0.27 | 1.95±0.39 | 1.81±0.35 |
| post1 | 2.13±0.16 | 2.14±0.16 | 2.75±0.22 | 2.05±0.29 | 1.86±0.37 | 1.69±0.28 |
| post2 | 2.07±0.21 | 2.13±0.22 | 2.65±0.25 | 1.97±0.36 | 1.99±0.28 | 1.74±0.31 |
| post3 | 2.15±0.21 | 2.15±0.22 | 2.75±0.22 | 1.96±0.25 | 1.79±0.29 | 1.72±0.27 |

| θ (4-8 Hz) iTBS | | | | | | |
| --- | --- | --- | --- | --- | --- | --- |
|  | MS A | MS B | MS C | MS D | MS E | MS F |
| pre | 1.61±0.27 | 1.81±0.19 | 1.85±0.25 | 2.05±0.31 | 1.44±0.27 | 1.56±0.27 |
| post1 | 1.63±0.23 | 1.73±0.22 | 1.88±0.31 | 1.95±0.32 | 1.41±0.32 | 1.51±0.34 |
| post2 | 1.59±0.23 | 1.71±0.21 | 1.81±0.28 | 77.71±9.19 | 65.31±8.35 | 69.59±4.71 |
| post3 | 1.61±0.18 | 1.69±0.25 | 1.95±0.22 | 1.96±0.31 | 1.41±0.26 | 1.41±0.27 |

| α (8-12 Hz) iTBS | | | | | | |
| --- | --- | --- | --- | --- | --- | --- |
|  | MS A | MS B | MS C | MS D | MS E | MS F |
| pre | 0.93±0.17 | 1.05±0.21 | 1.19±0.18 | 0.77±0.18 | 0.55±0.19 | 0.78±0.22 |
| post1 | 0.97±0.16 | 1.07±0.14 | 1.24±0.16 | 0.84±0.21 | 0.62±0.17 | 0.83±0.21 |
| post2 | 0.92±0.19 | 1.02±0.19 | 1.16±0.16 | 0.76±0.19 | 0.64±0.22 | 0.81±0.28 |
| post3 | 0.92±0.27 | 0.99±0.19 | 1.19±0.24 | 0.78±0.19 | 0.51±0.21 | 0.71±0.28 |

| β (15-30 Hz) iTBS | | | | | | |
| --- | --- | --- | --- | --- | --- | --- |
|  | MS A | MS B | MS C | MS D | MS E | MS F |
| pre | 2.26±0.25 | 2.21±0.34 | 2.54±0.48 | 1.57±0.29 | 0.81±0.31 | 1.81±0.49 |
| post1 | 2.26±0.26 | 2.19±0.22 | 2.55±0.31 | 1.61±0.25 | 0.91±0.36 | 1.76±0.29 |
| post2 | 2.23±0.31 | 2.18±0.28 | 2.57±0.44 | 1.53±0.34 | 0.94±0.41 | 1.69±0.37 |
| post3 | 2.24±0.33 | 2.14±0.29 | 2.53±0.32 | 1.55±0.27 | 0.86±0.28 | 1.67±0.36 |

| 𝛾 (30-40 Hz) iTBS | | | | | | |
| --- | --- | --- | --- | --- | --- | --- |
|  | MS A | MS B | MS C | MS D | MS E | MS F |
| pre | 2.69±0.49 | 2.27±0.41 | 3.11±1.05 | 1.09±0.32 | 0.69±0.48 | 1.96±0.68 |
| post1 | 2.58±0.36 | 2.35±0.27 | 3.14±0.82 | 1.09±0.25 | 0.74±0.43 | 1.89±0.45 |
| post2 | 2.56±0.44 | 2.29±0.37 | 3.05±0.69 | 1.15±0.32 | 0.91±0.73 | 1.82±0.46 |
| post3 | 2.49±0.62 | 2.34±0.57 | 3.02±0.73 | 1.06±0.32 | 0.72±0.33 | 1.81±0.58 |

| Broadband (1-40 Hz) sham | | | | | | |
| --- | --- | --- | --- | --- | --- | --- |
|  | MS A | MS B | MS C | MS D | MS E | MS F |
| pre | 2.04±0.61 | 1.93±0.46 | 2.91±0.58 | 1.72±0.53 | 1.26±0.41 | 1.35±0.51 |
| post1 | 2.09±0.56 | 1.89±0.46 | 2.78±0.48 | 1.68±0.54 | 1.21±0.37 | 1.37±0.54 |
| post2 | 2.11±0.61 | 1.84±0.44 | 2.89±0.54 | 1.72±0.59 | 1.26±0.37 | 1.34±0.58 |
| post3 | 1.94±0.56 | 1.97±0.39 | 2.81±0.48 | 1.61±0.57 | 1.18±0.43 | 1.31±0.47 |

| δ (1-4 Hz) sham | | | | | | |
| --- | --- | --- | --- | --- | --- | --- |
|  | MS A | MS B | MS C | MS D | MS E | MS F |
| pre | 2.21±0.33 | 2.01±0.38 | 2.84±0.51 | 1.87±0.55 | 1.84±0.26 | 1.61±0.42 |
| post1 | 2.31±0.32 | 1.93±0.33 | 2.78±0.41 | 1.84±0.51 | 1.86±0.33 | 1.73±0.36 |
| post2 | 2.31±0.35 | 1.91±0.31 | 2.79±0.53 | 1.91±0.56 | 1.83±0.27 | 1.65±0.34 |
| post3 | 2.18±0.35 | 2.03±0.29 | 2.72±0.35 | 1.91±0.45 | 1.87±0.34 | 1.65±0.31 |

*Cont.*

| θ (4-8 Hz) sham | | | | | | |
| --- | --- | --- | --- | --- | --- | --- |
|  | MS A | MS B | MS C | MS D | MS E | MS F |
| pre | 1.76±0.49 | 1.68±0.25 | 1.96±0.47 | 1.84±0.53 | 1.46±0.23 | 1.62±0.45 |
| post1 | 1.77±0.39 | 1.65±0.28 | 1.99±0.45 | 1.81±0.52 | 1.32±0.23 | 1.52±0.38 |
| post2 | 1.77±0.44 | 1.68±0.31 | 2.03±0.42 | 1.89±0.54 | 1.37±0.25 | 1.49±0.40 |
| post3 | 1.76±0.46 | 1.63±0.24 | 1.97±0.51 | 1.86±0.55 | 1.33±0.26 | 1.45±0.31 |

| α (8-12 Hz) sham | | | | | | |
| --- | --- | --- | --- | --- | --- | --- |
|  | MS A | MS B | MS C | MS D | MS E | MS F |
| pre | 0.95±0.21 | 0.97±0.15 | 1.28±0.23 | 0.74±0.32 | 0.59±0.21 | 0.73±0.27 |
| post1 | 1.00±0.24 | 0.96±0.21 | 1.25±0.19 | 0.74±0.27 | 0.58±0.17 | 0.79±0.29 |
| post2 | 0.98±0.27 | 0.96±0.22 | 1.22±0.16 | 0.71±0.28 | 0.61±0.15 | 0.81±0.31 |
| post3 | 0.92±0.24 | 1.04±0.18 | 1.21±0.21 | 0.72±0.26 | 0.55±0.19 | 0.75±0.25 |

| β (15-30 Hz) sham | | | | | | |
| --- | --- | --- | --- | --- | --- | --- |
|  | MS A | MS B | MS C | MS D | MS E | MS F |
| pre | 2.25±0.38 | 2.00±0.50 | 2.62±0.41 | 1.53±0.49 | 0.88±0.23 | 1.61±0.55 |
| post1 | 2.25±0.51 | 1.99±0.51 | 2.54±0.49 | 1.45±0.51 | 0.87±0.40 | 1.61±0.51 |
| post2 | 2.24±0.41 | 2.01±0.41 | 2.52±0.37 | 1.53±0.49 | 0.89±0.25 | 1.61±0.45 |
| post3 | 2.24±0.39 | 2.07±0.43 | 2.56±0.36 | 1.51±0.43 | 0.84±0.25 | 1.58±0.41 |

| 𝛾 (30-40 Hz) sham | | | | | | |
| --- | --- | --- | --- | --- | --- | --- |
|  | MS A | MS B | MS C | MS D | MS E | MS F |
| pre | 2.34±0.48 | 2.11±0.54 | 2.91±0.65 | 1.06±0.39 | 0.77±0.51 | 1.67±0.54 |
| post1 | 2.39±0.68 | 2.11±0.66 | 2.81±0.68 | 1.03±0.41 | 0.64±0.37 | 1.64±0.59 |
| post2 | 2.55±0.58 | 2.01±0.49 | 2.83±0.76 | 1.03±0.41 | 0.71±0.33 | 1.81±0.59 |
| post3 | 2.42±0.46 | 2.11±0.55 | 2.79±0.55 | 1.04±0.30 | 0.72±0.25 | 1.67±0.42 |
